# Hypofractionated SBRT versus conventionally fractionated EBRT for prostate cancer: comparison of PSA slope and nadir

**DOI:** 10.1186/1748-717X-9-42

**Published:** 2014-02-02

**Authors:** Mekhail Anwar, Vivian Weinberg, Albert J Chang, I-Chow Hsu, Mack Roach, Alexander Gottschalk

**Affiliations:** 1 Department of Radiation Oncology, University of California San Francisco, Helen Diller Comprehensive Cancer Center, 1600 Divisadero St. Suite H1031, Box 1708, San Francisco, CA 94143-1708, USA

**Keywords:** SBRT, Stereotactic body radiotherapy, Prostate, External beam, Conventionally fractionated, Nadir, Kinetics, Slope

## Abstract

**Background:**

Patients with early stage prostate cancer have a variety of curative radiotherapy options, including conventionally-fractionated external beam radiotherapy (CF-EBRT) and hypofractionated stereotactic body radiotherapy (SBRT). Although results of CF-EBRT are well known, the use of SBRT for prostate cancer is a more recent development, and long-term follow-up is not yet available. However, rapid post-treatment PSA decline and low PSA nadir have been linked to improved clinical outcomes. The purpose of this study was to compare the PSA kinetics between CF-EBRT and SBRT in newly diagnosed localized prostate cancer.

**Materials/methods:**

75 patients with low to low-intermediate risk prostate cancer (T1-T2; GS 3 + 3, PSA < 20 or 3 + 4, PSA < 15) treated without hormones with CF-EBRT (>70.2 Gy, <76 Gy) to the prostate only, were identified from a prospectively collected cohort of patients treated at the University of California, San Francisco (1997–2012). Patients were excluded if they failed therapy by the Phoenix definition or had less than 1 year of follow-up or <3 PSAs. 43 patients who were treated with SBRT to the prostate to 38 Gy in 4 daily fractions also met the same criteria. PSA nadir and rate of change in PSA over time (slope) were calculated from the completion of RT to 1, 2 and 3 years post-RT.

**Results:**

The median PSA nadir and slope for CF-EBRT was 1.00, 0.72 and 0.60 ng/ml and -0.09, -0.04, -0.02 ng/ml/month, respectively, for durations of 1, 2 and 3 years post RT. Similarly, for SBRT, the median PSA nadirs and slopes were 0.70, 0.40, 0.24 ng and -0.09, -0.06, -0.05 ng/ml/month, respectively. The PSA slope for SBRT was greater than CF-EBRT (p < 0.05) at 2 and 3 years following RT, although similar during the first year. Similarly, PSA nadir was significantly lower for SBRT when compared to EBRT for years 2 and 3 (p < 0.005).

**Conclusion:**

Patients treated with SBRT experienced a lower PSA nadir and greater rate of decline in PSA 2 and 3 years following completion of RT than with CF-EBRT, consistent with delivery of a higher bioequivalent dose. Although follow-up for SBRT is limited, the improved PSA kinetics over CF-EBRT are promising for improved biochemical control.

## Background

Patients with early stage prostate adenocarcinoma face a challenge in selecting from a variety of curative radiotherapy options, ranging from conventionally fractionated external beam radiotherapy (CF-EBRT), taking weeks, to single session brachytherapy implants. Increasingly, conformal radiation delivery techniques have safely escalated the dose to the prostate by either increasing the number of daily fractions [1-3] or adding a boost [4-7]. These approaches increase the delivered bioequivalent dose (BED), and consequently the biochemical control rate, but are associated with additional treatment time or, in the case of a brachytherapy boost, an invasive procedure.

The desire to deliver a higher bioequivalent dose (BED) to the prostate in fewer treatments prompted the investigation of hypofractionation, whereby the dose per fraction is increased while the total number of fractions is decreased. This strategy coupled well with reports of a low α/β ratio [8] (Table 1) for prostate cancer. Moreover, the reported α/β ratio values for prostate adenocarcinoma are consistently less than 3, indicating that prostate adenocarcinoma is a slow growing cancer with a greater ability to repair damage between fractions [9]. This implies that hypofractionated radiotherapy should have an increased biologic effect over standard fractionation. A review of hypofractionation trials demonstrated an increasing biochemical control rate with increasing BED, assuming an α/β of 1.5 [9].

**Table 1:** Summary of α/β values

**Ref**	**α/β (Gy)**	**95% Confidence interval**
Brenner and Hall [8]	1.5	[0.8,2.2]
Arcangeli 2010	-0.45	[-1.31, 0.41]*
Leborgne 2011[10]	1.86	[0.7, 5.1]
Lukka 2005[11]	2.02	[-1.03, 5.07]*
Valdagni 2005	7.44	[-13.97, 28.86]*
Yeoh 2011[12]	0.13	[-1.06, 1.31]*
Vogelius 2013 [13]	-0.07	[-0.73 – 0.59]
Williams 2007 [14]	2.6	[0.9, 4.8]
Fowler 2001 [15]	1.49	[1.25, 1.76]
Brenner 2002 [16]	1.2	[0.03, 4.1]

(*Taken from Vogelius et al. [13]).

These observations led to use of high dose rate brachytherapy (HDR), first as a boost [17-19], and then as definitive monotherapy [20-23]. Due to its superior conformality, HDR could deliver high bioequivalent doses while sparing neighboring critical structures. Although these studies leveraged hypofractionation, some patients are not operative candidates for HDR or wish to avoid an invasive procedure. More recently, the advent of image guided stereotactic body radiotherapy (SBRT) has allowed the precise delivery of highly conformal radiotherapy, enabling a high dose to be delivered to a target, while sparing nearby normal tissue, something previously only achievable with brachytherapy. Therefore, we have replicated the dosimetry of HDR (38 Gy in 4 fractions) using Stereotactic Body Radiotherapy (SBRT) [24], in effect emulating HDR via a non-invasive external beam approach.

Feasibility results for the use of hypofractionated SBRT in prostate cancer have been reported by our group [25], as well as others [26-28]. These early results are promising, though the application of SBRT for prostate adenocarcinoma is too recent a development to allow long term comparisons of efficacy with established treatment methods. However, the prevalence of this disease combined with the myriad of treatment options motivates further comparisons based on what data are currently available.

Prostate cancer is unique in that a well-established biomarker, prostate specific antigen (PSA), is available for monitoring response to treatment. In patients whose PSA level is not confounded by androgen deprivation therapy, analysis of PSA kinetics after treatment could reveal the biological effect of radiation on prostate cancer and potentially reflect clinical outcome. Lower PSA nadir and rapid decline in PSA after treatment have been related to improved clinical outcome. Specifically, a lower PSA nadir (< 0.5 ng/ml) has been associated with increased freedom from biochemical failure [29-32]. While increasing PSA values can either indicate biochemical failure [33] or PSA bounce [34], continued decline or stability in PSA is linked with biochemical control [35,36]. The interpretation of the PSA rate of decline following radiotherapy, as measured by the PSA slope, is more controversial. Some studies have shown a relationship between the magnitude of the PSA slope and clinical outcomes [35,37], while other studies have not [38-40]. Regardless, the rate of PSA decline is reflective of the cellular response to radiation. Theoretically, in patients whose PSA level is not confounded by the use of hormones or adenocarcinoma outside the radiation field, the study of PSA kinetics after treatment could yield information about the biological effect of radiation on prostate cancer and, potentially, clinical outcome.

We seek to gain insight into the potential long term clinical outcomes of SBRT for prostate cancer by comparing PSA kinetics (nadir and slope) in patients treated with SBRT with those of patients treated with CF-EBRT. Because the two modalities use the same method of delivering ionizing radiation, 6 MV photons, the major differences between the cohorts are the fraction size and total dose. Our hypothesis is that, due to the low reported α/β ratio for prostate cancer, the hypofractionated regimen delivered with SBRT, should produce a more substantial PSA response than CF-EBRT, as reflected in a greater rate of PSA decline and a lower PSA nadir.

## Patients and methods

### Patient selection

This single institution retrospective study was approved by our Committee on Human Research with a waiver of informed consent and was complaint with the Health Insurance Portability and Accountability Act.

Patients with biopsy proven prostate adenocarcinoma were seen in a multidisciplinary clinic and counseled on treatment options, including surgery and radiotherapy. Patients classified as low to low-intermediate risk (T1-T2 with GS 3 + 3, PSA < 20 or GS 3 + 4, PSA < 15) were eligible for treatment with radiation to the prostate and seminal vesicles only, without androgen deprivation therapy (ADT). Treatment was selected by the patient and physician. At the time of this analysis, of the options available, including permanent prostate implant (PPI), high dose rate brachytherapy, CF-EBRT with intensity modulated radiation therapy (IMRT) and SBRT, 71 patients have been treated with SBRT using the Cyberknife robotic radiosurgery/SBRT system (Accuray Incorporated, Sunnyvale, CA).

In order to accurately assess PSA kinetics in response to radiotherapy, patients were excluded if they failed therapy by the Phoenix definition [33], received pelvic radiotherapy, or had their PSA levels suppressed by the use of hormones. This was done to insure a uniform population in which to evaluate PSA outcomes. All included patients had at least 1 year of follow-up, and 3 serial PSAs. Patients were followed by ultrasensitive PSA assay, and results below the detection limit of the assay (for example < 0.1 ng/ml) were entered as the respective detection limit (for example 0.1 ng/ml for a value of < 0.1 ng/ml) for the purposes of data analysis. A PSA bounce was determined when the PSA value at follow-up increased over the previous nadir by > 0.2 ng/mL but subsequently steadily decreased with follow-up. Of the 71 eligible patients, 43 patients met these criteria. To identify the cohort of patients treated with CF-EBRT, the records from a prospectively collected cohort of patients treated at UCSF from 1997 through 2006 were reviewed. We identified 75 patients treated with standard fractionated EBRT who met the above inclusion criteria. Since the vast majority of patients treated with CF-EBRT alone received doses between 70.2 Gy and 76 Gy, we excluded patients who received doses above and below this interval in order to have a homogenous dose response in this group of patients. A comparison between the two radiotherapy cohorts of patient baseline characteristics is shown in Table 2.

**Table 2:** Patient baseline characteristics

			**CK**	**EBRT**
# Evaluable patients				
	Total		43	75
# Follow up from end of RT				
		Thru Year 1	43	42
		Thru Year 2	38	62
		Thru Year 3	27	68
# with PSA follow-up for all 3 intervals			26	37
Median age at RT (yrs) (range)			69.0 (51 – 83)	69.8 (55 – 82)
Gleason score:				
	3 + 3		24 (56%)	59 (79%)
	3 + 4		19 (44%)	16 (21%)
Pretreatment PSA (ng/mL)				
	Median (range)		6.2 (2.0 – 13.5)	5.9 (0.1 – 16.7)
	# >10.0 ng/mL		10 (23%)	19 (25%)
Years of RT			2006 – 2011	1997 – 2006
Median follow-up (mos.) (range)			29.3 (12 – 75)	62.1 (15 – 156)

### SBRT technique

The specifics of the SBRT technique have been described previously [25], but briefly, the dose and fractionation are based on the UCSF HDR monotherapy experience, with 38 Gy in 4 fractions of 9.5 Gy to an isodose of approximately 60-80% and a 2 mm expansion for patient setup and motion. Prior to treatment, 3 fiducial markers were inserted into the prostate, enabling real-time tracking of and automatic beam adjustment for intrafraction prostate motion. No patients were treated with hormone therapy or pelvic radiotherapy.

### Statistical analysis

Because the use of SBRT for prostate adenocarcinoma is a relatively new application, significantly longer follow-up exists for patients treated with CF-EBRT as opposed to SBRT, preventing direct comparison of clinical outcomes. To eliminate the effect of differing follow-up durations between the groups, we calculated the PSA nadir and rate of change in PSA over an interval of time from the completion of radiotherapy (RT) to 1, 2, and 3 years post-treatment with the requirement of at least 3 PSA measurements recorded for each estimate. It might mean that a patient has only 2 measurements during years 1 and 2 after radiotherapy so this patient would be evaluated for the two year interval with the slope and PSA nadir determined from 4 values. Of the eligible patients, 37 and 26 patients treated with EBRT and SBRT, respectively, had increasing PSA follow-up over the 3 years with a sufficient number of measurements to calculate the slope and determine the PSA nadir for each of the three intervals.

To summarize the PSA measurements the slope, the rate of change of PSA over time, with units of ng/ml/month, the PSA nadir and time to PSA nadir were calculated for each of three intervals from RT. Descriptive statistics (e.g. mean, median, range) were tabulated for patient, disease and PSA parameters for each RT cohort. A *t* test was performed to compare mean values, the Mann–Whitney test was used to compare distributions and the log rank test was calculated to compare the distributions of time to PSA nadir between the two RT subsets. There was no adjustment for multiple comparisons and statistical significance was defined as a probability value less than 0.05. Analyses were performed using Statistica v6.0 (StatSoft, Inc, Tulsa, OK).

## Results

**Figure 1:**
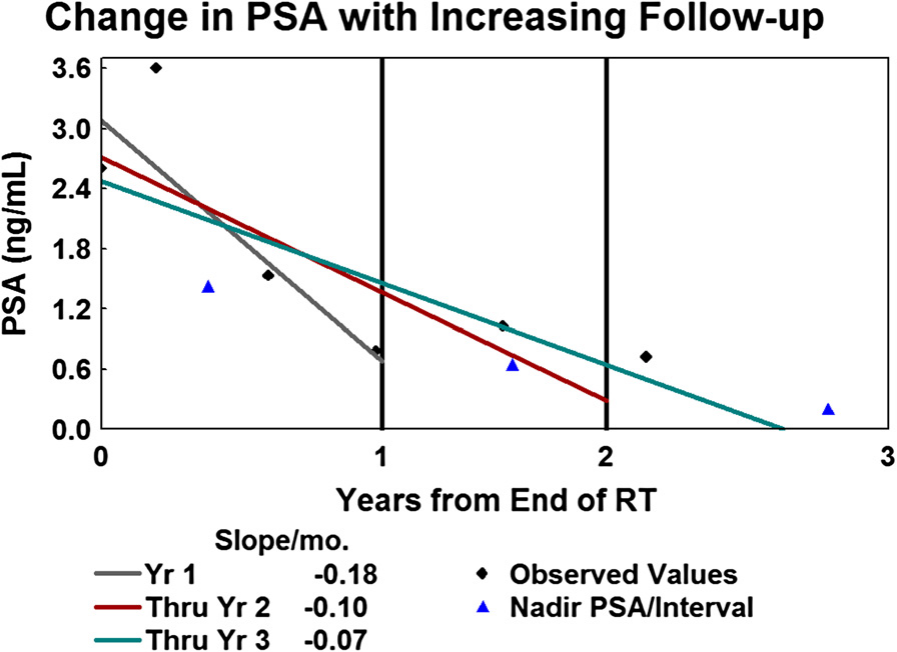
Calculation of slope and PSA nadir for a sample patient for 3 time durations, 1, 2 and 3 years post-RT for a patient treated with SBRT.

To investigate PSA kinetics after radiotherapy, the slope and nadir of the PSA outcome after radiotherapy was calculated for each radiotherapy cohort for 3 intervals following radiotherapy (0 to 1 year, 0 to 2 years, and 0 to 3 years, Table 3). Figure 1 is an illustrative example for a single patient treated with SBRT with serial PSA values declining over time, with the rate of change in PSA since radiotherapy summarized by the slope for each of the 3 time intervals. The rate of PSA decline (slope) for the CF-EBRT cohort was maximal in the first year, but tapered off quickly in the following years, with median values of -0.09, -0.04, -0.02 ng/ml/month for durations of 1, 2 and 3 years post RT, respectively. Consistent with a slower rate of decline in years 2 and 3 for this cohort, the PSA nadir did not continue to substantially drop after year 2, going from a median of 1.00 ng to 0.72 ng to 0.60 ng for durations of 1, 2 and 3 years post RT, respectively. Although the magnitude of the slopes for both SBRT and CF-EBRT decreased with time, there was a greater prolonged rate of decline with SBRT. While the distribution of the slope for SBRT initially did not differ from the CF-EBRT group in year 1 (medians: -0.09 ng/ml/month for both groups), the distributions were significantly different with a greater median rate of change for 2 and 3 years post-RT (-0.06 and -0.05 ng/ml/month, respectively for SBRT versus (-0.04 and -0.04 ng/ml/month for CF-EBRT). This trend was also seen when analysis was limited to those patients with 3 years of follow up, although it was no longer statistically significant in this reduced patient population. The steeper rate of PSA decline for SBRT resulted in lower median PSA nadirs of 0.70, 0.40, and 0.24 ng for durations of 1, 2 and 3 years post RT which was statistically significantly lower for years 2 and 3 (p ≤ 0.002). Achieving a significantly lower PSA nadir 2 and 3 years after RT was also observed when limited to patients with more complete PSA follow-up each year (p ≤ 0.02, Table 4). Consistent with a lower PSA nadir for SBRT, the time to PSA nadir was statistically longer for SBRT when compared to CF-EBRT over 3 years (p < 0.005). The incidence of PSA bounce was more frequent in patients treated with either SBRT (5 patients, 12%) compared to CF-EBRT (7 patients, 9%), but the small number of instances prevents determination of statistical significance.

**Table 3:** Results (all patients)

			**SBRT**	**CF-EBRT**	**p-value**
		Through year			
PSA Measurements ^#^					
	Mean (range)	1	3.9 (2 – 6)	4.1 (3 – 11)	
		2	5.8 (4 – 9)	5.6 (3 – 15)	
		3	7.6 (5 – 11)	7.3 (3 – 21)	
Nadir PSA (ng/mL)					
	Median (range)	1	0.70 (0 – 2.5)	1.00 (0 – 8.5)	
		2	0.40 (0 – 1.4)	0.72 (0 – 2.7)	p = 0.0005*
		3	0.24 (0.1 – 1.4)	0.60 (0 – 2.2)	p = 0.002*
Time to Nadir PSA (mos.)					
	Median (range)	1	12.0 (2.7 – 15.0)	11.5 (1.2 – 15.0)	
		2	21.0 (2.7 – 26.9)	18.0 (1.2 – 26.9)	
		3	32.3 (2.7 – 41.6)	28.6 (1.0 – 41.1)	p = 0.004^
Rate of PSA change: ng/mL/month					
	Median slope (range)	1	-0.09 (-0.88, 0.04)	-0.09 (-0.60, 0.06)	
		2	-0.06 (-0.38, 0.01)	-0.04 (-0.65, 0.05)	p = 0.04*
		3	-0.05 (-0.19, 0.00)	-0.02 (-0.38, 0.04)	p = 0.006*

SBRT: Stereotactic Body Radiotherapy.

CF-EBRT: Conventionally fractionated external beam radiotherapy.

p: Statistical significance.

# The *t* test was performed to compare the mean number of PSA measurements between the two groups for each interval with means and ranges presented to summarize the results.

^ The log rank test was used due to compare the time to nadir PSA distributions between the two groups for each interval with medians and ranges presented to summarize the results.

* The Mann–Whitney test was performed to compare the PSA nadir and slope distributions between the two groups with medians and ranges presented to summarize the results.

**Table 4:** Results (Patients with continuous PSA follow up in all 3 intervals)

			**SBRT**	**CF-EBRT**	**p-value**
		Through year			
# Evaluable patients with PSA			26	37	
Follow-up for All 3 intervals					
PSA measurements					
	Mean (range)	1	3.8 (2 – 5)	4.2 (3 – 11)	
		2	5.8 (4 – 8)	6.6 (4 – 15)	
		3	7.6 (5 – 11)	9.0 (6 – 21)	p = 0.03#
Nadir PSA (ng/mL)					
	Median (range)	1	0.81 (0.1 – 2.5)	1.00 (0 – 8.5)	
		2	0.45 (0.1 – 1.4)	0.86 (0 – 2.5)	p = 0.02*
		3	0.25 (0.1 – 1.4)	0.70 (0 – 2.2)	p = 0.002*
Time to Nadir PSA (mos.)					
	Median (range)	1	12.0 (2.7 – 15.0)	11.5 (1.2 – 15.0)	
		2	22.1 (2.7 – 26.9)	17.9 (1.2 – 26.9)	
		3	31.5 (2.7 – 41.6)	27.4 (1.2 – 41.0)	p = 0.02^
Rate of PSA change: ng/mL/month					
	Median slope (range)	1	-0.08 (-0.88, 0.04)	-0.09 (-0.60, 0.06)	
		2	-0.06 (-0.38, 0.004)	-0.04 (-0.65, 0.05)	
		3	-0.05 (-0.19, 0.001)	-0.03 (-0.38, 0.02)	

SBRT: Stereotactic Body Radiotherapy.

CF-EBRT: Conventionally fractionated external beam radiotherapy.

p: Statistical significance.

# The *t* test was performed to compare the mean number of PSA measurements between the two groups for each interval with means and ranges presented to summarize the results.

^ The log rank test was used due to compare the time to nadir PSA distributions between the two groups for each interval with medians and ranges presented to summarize the results.

* The Mann–Whitney test was performed to compare the PSA nadir and slope distributions between the two groups with medians and ranges presented to summarize the results.

Although the inclusion criteria make this a relatively homogenous population and subsets for comparison of the PSA kinetics were small, the analysis by Gleason score shows that SBRT results in a lower PSA nadir regardless of Gleason score (3 + 3 versus 3 + 4, Table 5). Furthermore, these trends held constant when analyzed by age (Table 5) or pre-treatment PSA (Table 6).

**Table 5:** Comparison of PSA kinetics between CK and CF-EBRT by Gleason score and age subsets: probability values

		**Gleason score 3 + 3**	**Gleason score 3 + 4**
	Through year:		
Nadir PSA (ng/mL)			
	1	ns	ns
		p = 0.01	p = 0.03
		p = 0.03	p = 0.02
Time to Nadir PSA (mos.)			
	1	ns	ns
	2	ns	ns
	3	p = 0.02	ns
Rate of PSA change: ng/mL/month			
	1	ns	ns
	2	p = 0.02	ns
	3	p = 0.01	ns
		**Age < 70**	**Age ≥ 70**
Nadir PSA (ng/mL)			
	1	ns	ns
	2	p = 0.002	p = 0.03
	3	p = 0.01	ns
Time to Nadir PSA (mos.)			
	1	ns	ns
	2	ns	ns
	3	p = 0.004	ns
Rate of PSA change: ng/mL/Month			
	1	ns	ns
	2	ns	ns
	3	(p = 0.09)	p = 0.03

For each Gleason score subset (3 + 3 vs 3 + 4) and age subset (<70 vs ≥70) the PSA outcomes were compared between SBRT and CF-EBRT. The distributions for PSA nadir and PSA slope were compared using the Mann–Whitney test. The distributions for the time to PSA nadir was compared using the log rank test. [ns: not significant].

**Table 6:** Comparison of PSA kinetics between SBRT and CF-EBRT by pre-treatment PSA (probability values)

			**Pretreatment PSA (ng/mL)**	
		**≤10**	**>10**	**>4**
	Through year			
Nadir PSA (ng/mL)				
	1	ns	ns	ns
	2	p = 0.001	ns	p = 0.002
	3	p = 0.005	ns	p = 0.001
Time to Nadir PSA (mos.)				
	1	ns	ns	ns
	2	ns	ns	ns
	3	p = 0.04	p = 0.03	p = 0.01
Rate of PSA change: ng/mL/Month				
	1	ns	ns	ns
	2	p = 0.05	ns	(p = 0.08)
	3	(p = 0.06)	ns	p = 0.02

For each of the 3 pretreatment PSA subsets the PSA outcomes were compared between SBRT and CF-EBRT. The distributions for PSA nadir and PSA slope were compared using the Mann–Whitney test. The distributions for the time to PSA nadir was compared using the log rank test.

## Discussion

SBRT delivered in 4 fractions of 9.5 Gy has a BED of 218 Gy, assuming an α/β of 2 (e.g. BED2), compared with a BED2 of 140–150 Gy with CF-EBRT given in 35–38 fractions of 2 Gy. Consistent with dose escalation trials that have showed a lower PSA nadir with increased number of fractions [30,41,42] (and consequently increased BED), we expect the hypofractionated SBRT regimen to produce a lower PSA nadir as well as a greater rate of decline in PSA over the same time period, than CF-EBRT. Several reports have shown PSA kinetics after CF-EBRT to follow an exponential decay, with half-lives varying from 0.27 to 0.67 years [35,43,44], indicating that the majority of the PSA decline occurs in the first year, consistent with our results. Although both modalities have similar PSA slopes through year 1, the rate of PSA decline for patients treated with CF-EBRT substantially falls off, and approaches 0 at year 3. The rate of decline for those treated with SBRT does not fall off as quickly, and the rate of decline at 3 years is still notable. Although this cannot be used to derive clinical outcomes, it is clear that the hypofractionated regimen has a distinct effect on the prostate cancer cells which produce the predominance of the PSA. More importantly, the PSA nadirs for SBRT are statistically significantly lower than CF-EBRT for years 2 and 3, regardless of whether all eligible patients, or only those with long term follow up post-RT, are analyzed, consistent with delivery of a greater BED. Our findings are consistent with the trends reported by other investigators, with Zelefksy et al. [45] showing a PSA nadir of 0.6 ng/ml at 23 months with 81 Gy CF-EBRT, while Katz et al. [46] showed a longer, continued drop in PSA resulting in a PSA dropping to 0.12 ng/ml with patients treated with SBRT. Additionally, a pooled analysis of SBRT patients [47] further supported a trend with a more rapid drop in PSA, reporting a median PSA of 0.2 ng/ml at 3 years.

This study has several limitations. As previously noted, due to the large difference in follow-up between the SBRT and CF-EBRT study populations, overall PSA nadir could not be directly compared. Therefore, we analyzed the PSA nadir and slope in identical time intervals from completing RT for both groups, negating the effect of different follow up periods. Although prostate adenocarcinoma treated with CF-EBRT is known to reach its nadir in 2–3 years, the time to nadir is unknown for SBRT. Therefore, the median nadir during 3 years post RT of 0.24 ng/mL may not represent the true PSA nadir. In fact, the significant difference in the distributions of the slope which is still present through years two and three may indicate the occurrence of a lower PSA nadir with increased follow-up. The significantly lower PSA nadir for SBRT versus CF-EBRT is also reflected in the longer time to nadir for SBRT. Continued follow-up will allow for additional monitoring of PSA slope and nadir in SBRT patients and to investigate these trends. Additionally, in order to accurately compare the PSA kinetics, patients who failed by the Phoenix definition were excluded. Patients with a rising PSA after treatment may represent those with microscopic foci of prostate adenocarcinoma outside the prostate or those with high grade disease not adequately represented in the biopsy.

The majority of the included patients receiving CF-EBRT here were treated in the pre-dose escalation era, and received between 70.2 and 76 Gy. This represents only an 8% difference in BED2 from a dose of 78 Gy, so while these lower prescription doses may decrease both the magnitude of the PSA slope and nadir for this group of patients, the effect should not be significant when compared to modern doses. Although the inclusion criteria were limited to patients with low to low-intermediate risk prostate adenocarcinoma, differences in Gleason score and pre-treatment PSA may represent variations in the underlying biology of the prostate adenocarcinoma, and therefore affect the response to radiation. To address this, a comparison between the RT cohorts in PSA kinetics within subsets was performed, and patients treated with SBRT had lower PSA nadir regardless of Gleason score, pre-treatment PSA (≤10, >4), or age < 70.

## Conclusion

Delivery of increased BED in standard fractionation has been associated with lower PSA nadir and improved biochemical outcome in patients treated for prostate adenocarcinoma. Studies of the α/β ratio for prostate cancer point to a low value that would benefit from hypofractionated treatment. Not only should a hypofractionated regimen provide increased BED and biochemical control, the need for far fewer fractions means a dramatically decreased treatment time for the patient. Existing studies using high dose rate brachytherapy as monotherapy for low and low-intermediate risk patients have demonstrated success, confirming this approach, but require an invasive procedure. To leverage the benefits of hypofractionation while still utilizing external beam radiotherapy, at UCSF we have been treating low and low intermediate risk prostate cancer patients with SBRT in a dose and fractionation scheme modeled from the HDR experience, 9.5 Gy for 4 fractions, providing a BED2 of 218 Gy. We hypothesized that the greater BED delivered by SBRT would result in improved kinetics (consistently greater rate of PSA decline over time and lower PSA nadir). The data presented here support this, with lower PSA nadirs as well as a steeper decline in PSA from SBRT greater in years 2 and 3 after treatment than with CF-EBRT. These findings are consistent with the notion of a low α/β for prostate cancer and significantly increased BED with hypofractionation. Although follow-up for SBRT is limited due to its recent introduction into the clinic, the improved PSA kinetics of SBRT over CF-EBRT are promising for improved biochemical control.

## Abbreviations

SBRT: Stereotactic body radiotherapy; CF-EBRT: Conventionally fractionated external beam radiotherapy.

## Competing interests

The authors declare that they have no competing interests.

## Authors’ contributions

MA conceived of the study, and participated in its design and coordination and helped to draft the manuscript. VW participated in the design of the study, performed the statistical analysis and helped to draft the manuscript. AJC participated in its design and coordination and helped to draft the manuscript. ICH participated in its design and coordination and helped to draft the manuscript. MR participated in its design and coordination and helped to draft the manuscript. AG conceived of the study, and participated in its design and coordination and helped to draft the manuscript. All authors read and approved the final manuscript.
